# Loss of type III collagen has limited effect on tendon healing in young adult mice

**DOI:** 10.1016/j.matbio.2026.102012

**Published:** 2026-05-06

**Authors:** Margaret K. Tamburro, Jaclyn A. Carlson, William K. Yen, Stephanie N. Weiss, Emma E. Kroll, Miranda K. Doro, Courtney A. Nuss, Mengcun Chen, Daniel C. Stewart, Jeremy D. Eekhoff, Louis J. Soslowsky, Susan W. Volk

**Affiliations:** a McKay Orthopaedic Laboratory, University of Pennsylvania, Philadelphia, PA, USA; b Department of Bioengineering, School of Engineering and Applied Sciences, University of Pennsylvania, Philadelphia, PA, USA; c Department of Clinical Sciences & Advanced Medicine, School of Veterinary Medicine, University of Pennsylvania, Philadelphia, PA, USA

**Keywords:** Type III collagen, Tendon, Injury, Healing, Scar, Biomechanics

## Abstract

Tendon injuries account for considerable and escalating clinical burden. After injury, tendons heal poorly with persistent fibrovascular scar. Unlike healthy tendon which is type I collagen (COL1)-rich and highly-aligned, fibrovascular scar is type III collagen (COL3)-rich and disorganized; this compositional and organizational change has been implicated in the functional deficits resulting from tendon injury. Accordingly, COL3 is historically considered a primary driver of poor tendon healing. Given compelling evidence of COL3’s role in regulating matrix structure and cell behavior, we sought to define the role of COL3 in driving physiologic/pathologic tendon healing through regulation of all phases of healing. Leveraging a mouse model of global, inducible *Col3a1* knockdown, we reduced *Col3a1* expression at the time of patellar tendon injury and investigated extracellular matrix, cellular, and mechanical changes at 1-, 3-, and 6-weeks post-injury. Based on data from other tissues, we hypothesized that COL3 loss would exacerbate poor tendon healing, with temporally-distinct contributions to tendon healing outcomes resulting from COL3’s critical regulation of matrix organization, cell phenotype and activities, and tissue mechanics. Unexpectedly, reduction of COL3 in young adult mice did not change collagen architecture and had few, nuanced impacts on cell behavior and tissue mechanics throughout healing. These findings challenge the conventional paradigm that COL3 drives poor tendon healing outcomes and emphasize the need to identify matrix and cell mechanisms contributing to poor tendon healing. Understanding COL3’s role in other injury models and lifespan contexts, including tendon development and aging, will provide more insight into its regulatory and therapeutic potential.

## Introduction

Tendon injuries heal poorly, leading to persistent pain and quality of life deficits that account for substantial and growing healthcare burden [1–4]. Healthy tendon matrix is primarily comprised of highly-aligned type I collagen (COL1) which endows high tensile strength. In contrast, type III collagen (COL3), which is deposited after injury in a wide variety of tissues, is the primary structural component of tendon scar tissue during healing [5–11]. A COL3-rich matrix is associated with decreased fibrillar collagen alignment and density in other tissues [12–14] as well as poor tensile mechanics [15]. Therefore, increased COL3 is classically accepted as a driver of poor tendon healing outcomes.

In conjunction with the well-characterized, sustained increase in COL3 content following tendon injury, an extensive and growing body of evidence implicates COL3 as a key regulator of matrix and cell behavior during each phase of canonical tendon healing: the inflammatory, proliferative, and remodeling phases. During the initial, inflammatory phase of tendon healing, *Col3a1* expression increases over 5-fold relative to pre-injury levels to establish a COL3-rich matrix within the acute tendon injury region [16]. Concurrently, immune cells migrate to the wound site to micro-debride injured tissue and recruit reparative cells [11,17–21], a process COL3 regulates in other contexts [22,23]. During the subsequent proliferative phase, recruited reparative cells multiply and further increase deposition of a COL3-rich, disorganized matrix. Activation, proliferation, and anabolism of fibroblasts are the defining cellular activities of the proliferative phase [24]. Notably in skin, myofibroblast activation, evidenced by alpha smooth muscle actin (αSMA) expression, is negatively regulated by COL3 [12,25]. As healing progresses to the remodeling phase, the established COL3-rich matrix is partially replaced with a more highly-aligned collagen matrix characterized by a progressive increase in COL1:COL3 content through coordinated matrix degradation and deposition. Contemporaneously, there is a critical shift in macrophage polarization from predominantly M1 (pro-inflammatory) to M2 (anti-inflammatory) macrophage phenotype [21,26,27], a process COL3 regulates in other tissues [22], which coincides with and supports increased matrix turnover. Importantly, while the dynamics of COL3 after tendon injury are well characterized, the specific mechanistic impacts of COL3 on both matrix and cells and their corresponding contributions to the relatively poor healing outcomes in tendon have not been established. Leveraging the interdependent and overlapping phases of tendon healing facilitates temporal discernment of mechanistic impacts of early COL3 loss on tendon healing.

Accordingly, our objective was to define the regulatory roles of COL3 throughout tendon healing by reducing *Col3a1* expression at the time of tendon injury and investigating consequential matrix, cell, and mechanical changes at early, middle, and late phases of tendon healing. We hypothesized that COL3 would have critical, temporally-distinct regulatory roles throughout tendon healing. Because COL3 is the primary structural component of the provisional healing matrix, we hypothesized that COL3 would be responsible for establishing early matrix structure to allow for inflammatory cell migration and transitional mechanical integrity during early healing. According to our data in other tissues, we expected that as healing progresses, COL3 would be important for regulating matrix density and alignment to support migration, proliferation, and activation of reparative cells [12,25]. Finally, in late healing, we predicted COL3 would drive persistent matrix disorganization, pathologic cell phenotype, and compromised mechanical integrity. Collectively, we hypothesized that COL3 loss at the time of injury would be detrimental to the overall process of tendon healing due to failure to form an early healing microenvironment that supports cell recruitment and activation leading to poor mechanical outcomes.

## Results

### ROSA CreERT2 driver facilitated efficacious, temporally-targeted reduction of col3a1 expression during tendon healing

**Fig. 1.**
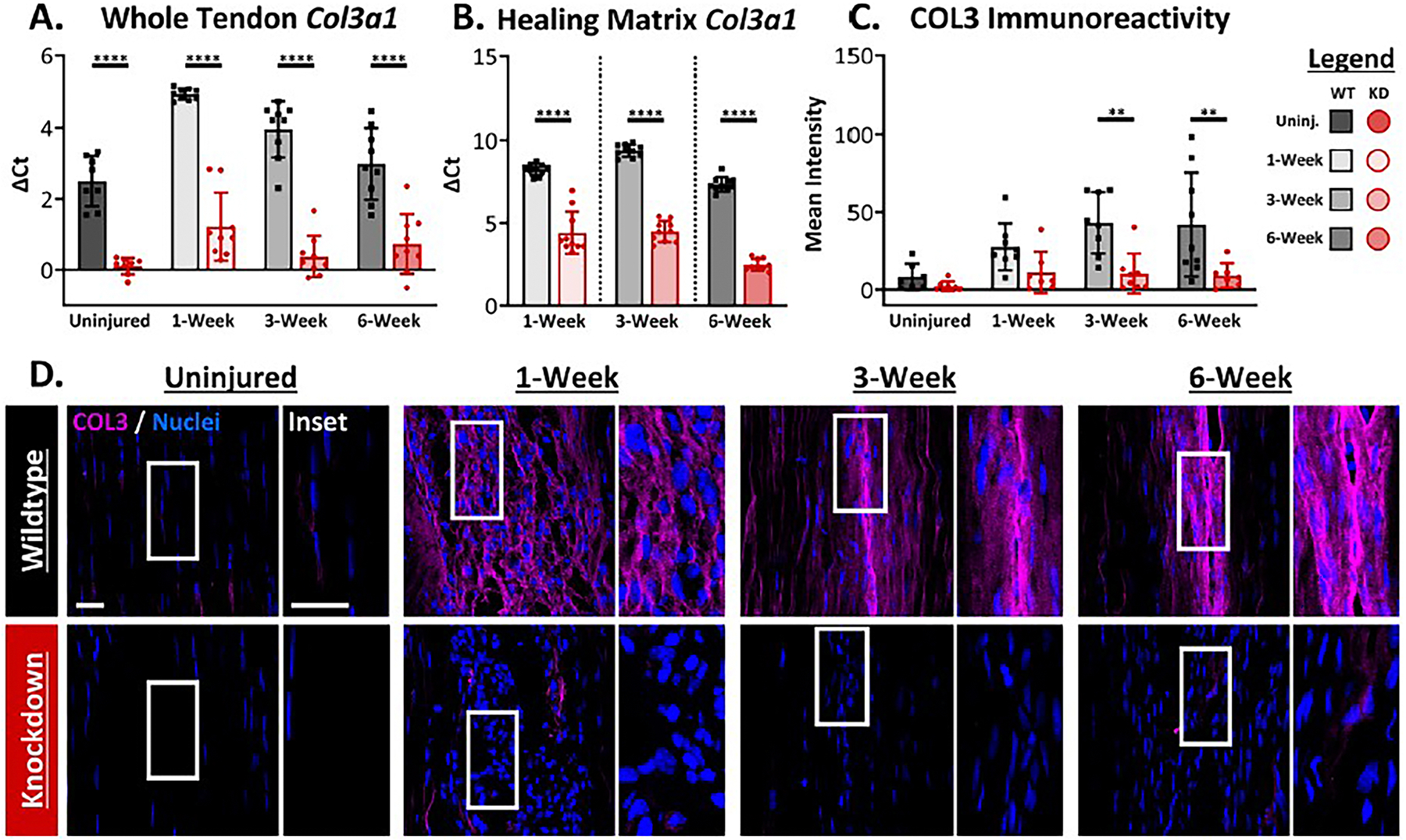
Tamoxifen-induced ROSA CreERT2 *Col3a1* excision reduced *Col3a1* expression and COL3 immunoreactivity in healing tendon. Compared to wildtype (WT) tendons, whole tendon *Col3a1* expression was reduced by tamoxifen injection in uninjured as well as 1-, 3-, and 6-week post-injury knockdown (KD) tendons (A). Additionally, *Col3a1* expression by cells in the healing matrix was reduced in the knockdown groups at all investigated timepoints (B). COL3 protein content, assessed with immunolocalization, demonstrated robust reduction at 3- and 6-weeks post-injury in knockdown groups (C, representative images shown in D). * p < 0.05, ** p < 0.01, *** p < 0.001, and **** p < 0.0001 for multiple comparison test (A,C) or *t*-test comparing genotypes at each timepoint (B). Scale bars are 25 μm, and quantitative data comes from regions of interest the size of representative insets. n ≥ 4 per timepoint per genotype per sex for whole tendon gene expression, n = 5 per timepoint per genotype per sex for healing matrix gene expression, and n ≥ 3 per timepoint per genotype per sex for immunolocalization.

As expected for wildtype tendons, whole tendon *Col3a1* expression increased after acute tendon injury (p < 0.0001 for main effect of timepoint). After tamoxifen injection, *Col3a1* knockdown tendons had substantially reduced *Col3a1* expression (80–90% knockdown efficiency) in the uninjured state and at all healing timepoints for assessment of both whole tendon and healing matrix (Fig. 1A-B). Reduction of *Col3a1* expression was consistent between biological sexes (Supplemental Table 5). Immunolocalization of COL3 further corroborated reduction of COL3 protein levels at 3- and 6-weeks post-injury (Fig. 1C-D). Importantly, knockdown of COL3 did not produce any qualitative changes in gait, physical activity, or other behavior.

*Col3a1* knockdown did not affect expression of *Col1a1* (not pre-amplified) or *Col1a2* in the healing matrix at any timepoint (Supplemental Figure 2A-B). Assessment of gene expression for other fibrillar collagens in the healing matrix revealed timepoint- and sex-specific changes. For mixed-sex analysis, compared to the wildtype healing tendons, *Col2a1* and *Col5a1* expression was increased at 3-weeks post-injury in *Col3a1* knockdown tendons, while *Col11a1* expression was decreased at 1-week post-injury (Supplemental Figure 2C-E). At 3-weeks post-injury, *Col5a1* and *Col11a1* expression were increased in female tendons and *Col1a2* expression was decreased in male tendons (Supplemental Table 6).

### Reduced Col3a1 expression during tendon healing did not alter collagenous matrix structure

**Fig. 2.**
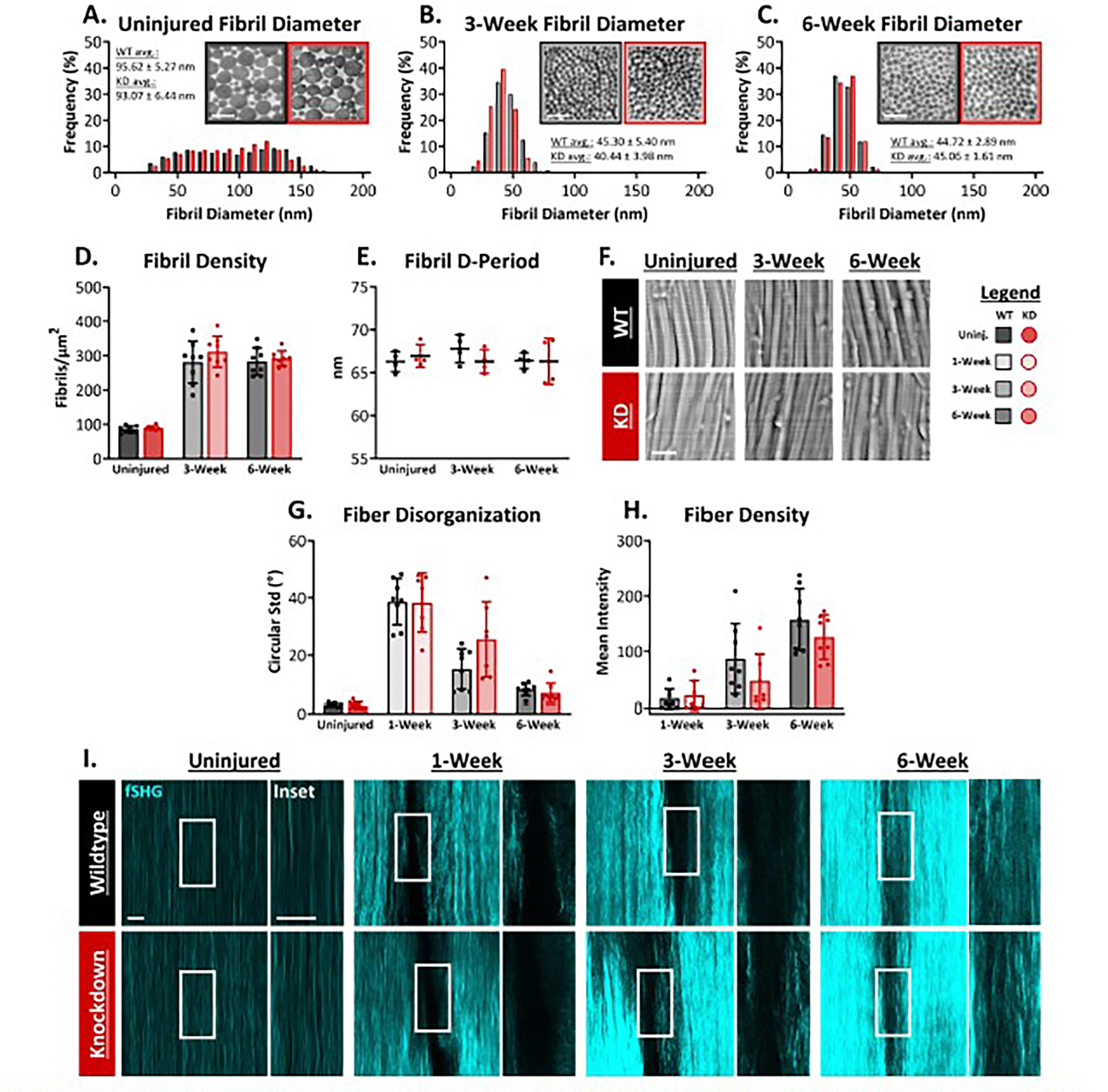
Collagen matrix nano- and microstructure was not influenced by *Col3a1* knockdown. Average fibril diameter in knockdown (KD) compared to wildtype (WT) tendons was not changed in the uninjured (A), 3-week (B), or 6-week (C) groups. Fibril density was also unaffected by *Col3a1* loss at all timepoints (D). Fibril D-period measured using atomic force microscopy was not affected by either genotype or timepoint (E, representative images shown in F). At the fiber level, circular standard deviation of fiber organization, which is inversely proportional to matrix alignment, was unaltered by *Col3a1* reduction at all stages of healing (G). Fiber density, indicated by the intensity of the forward second harmonic generation (fSHG) signal, was unaltered by *Col3a1* knockdown at all timepoints (H, representative images shown in I). Uninjured and injured tendon fibers were imaged with different laser intensities due to substantial differences in matrix density. Scale bars are 200 nm for fibril diameter images, 500 nm for fibril D-period images, and 25 μm for fiber images, and quantitative fiber data comes from regions of interest the size of representative insets. n = 4 per timepoint per genotype per sex for fibril diameter and density, n = 2 per genotype per sex for fibril D-period, and n ≥ 3 per timepoint per genotype per sex for fiber properties.

Effects of *Col3a1* knockdown on collagenous matrix structure in healing tendons were investigated at the nanoscale and the microscale to understand fibril and fiber changes, respectively. At the nanoscale, fibril diameter in uninjured tendons demonstrated expected bimodal distributions, but comparing average fibril diameter did not demonstrate differences in knockdown compared to wildtype tendons (Fig. 2A). Likewise, neither female nor male uninjured knockdown tendons had different average fibril diameter than their wildtype counterparts (Supplemental Figure 3A, D). After injury, fibril diameters in the healing matrix were unimodal, reflecting deposition of new, immature fibrils. Average fibril diameter for tendons in knockdown compared to wildtype groups was not changed at 3-weeks or 6-weeks post-injury (Fig. 2B-C), and this was consistent for both female and male tendons (Supplemental Figure 3B-C, E-F). Likewise, fibril density was not changed by *Col3a1* knockdown in uninjured or healing samples (Fig. 2D), and fibril D-period was also not affected by genotype and did not vary across healing timepoint (Fig. 2E-F). This lack of effect of COL3 on fibril density and D-period held true across sexes (Supplemental Table 7).

Fiber changes were similarly unremarkable. Matrix disorganization was measured as the circular standard deviation of fiber orientation, where a higher value indicates more matrix disorganization. Uninjured tendons had low matrix disorganization as expected. After injury, matrix disorganization was increased (p < 0.0001 for main effect of timepoint). However, reduction of *Col3a1* expression did not influence matrix organization in uninjured or healing tendons (Fig. 2G, I). Fibrillar collagen density, measured as the mean intensity of the forward second harmonic generation (fSHG) signal, increased throughout healing (p < 0.0001 for main effect of timepoint) but did not vary between knockdown and wildtype groups at any healing timepoint (Fig. 2H-I). Matrix density was not comparable between uninjured and injured tendons because substantial differences in fiber density required different laser intensities for fSHG imaging. The paucity of *Col3a1* expression-based fiber differences was consistent between female and male tendons (Supplemental Table 7). Ultimately, reduced *Col3a1* expression did not affect collagenous matrix structure or density at either the nano- or microscale.

### Reduced Col3a1 expression minimally impacted stromal cell dynamics during tendon healing

**Fig. 3.**
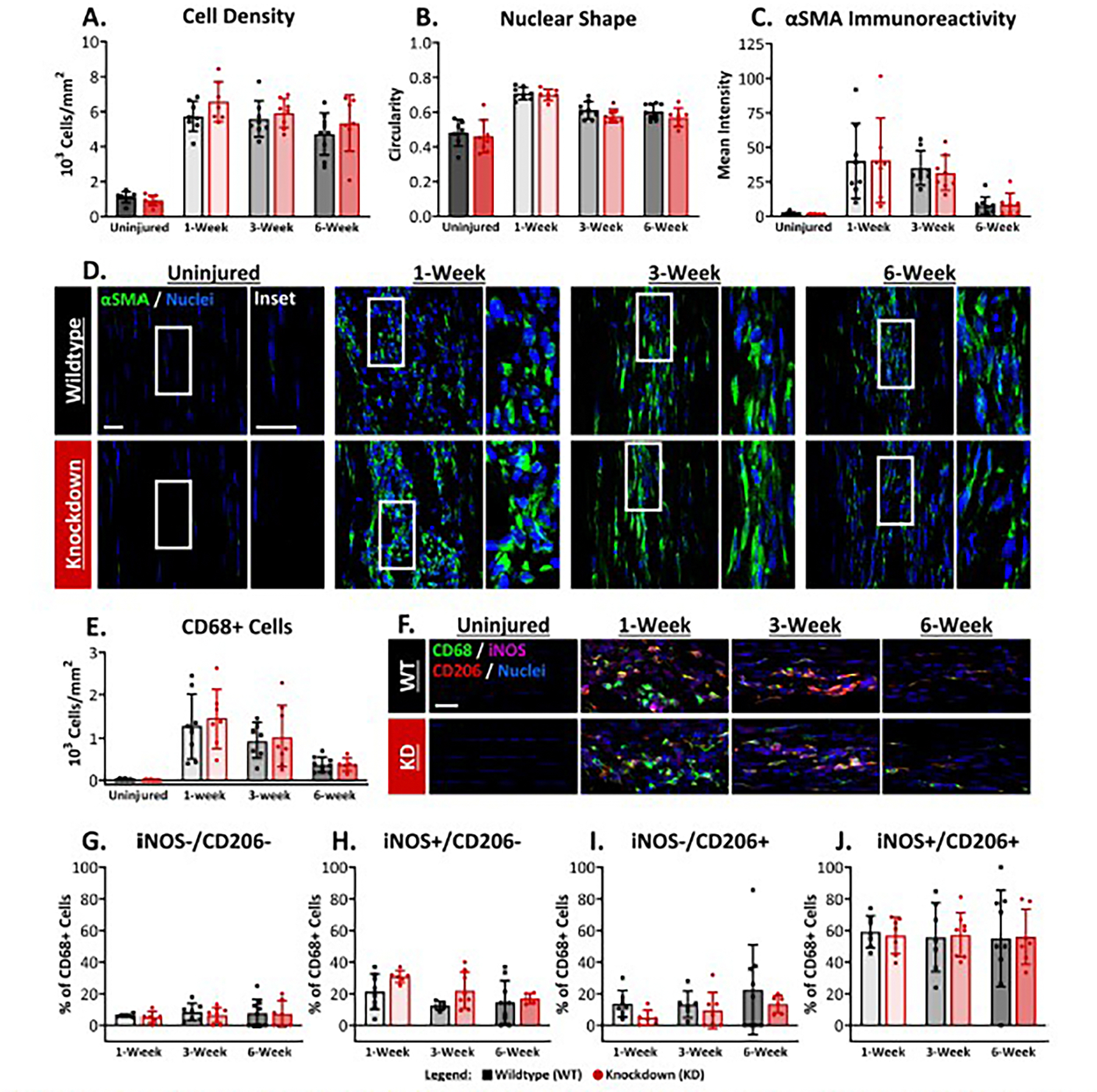
Cellular composition in healing tendons was not markedly influenced by *Col3a1* knockdown. Tendon cell density (A) and nuclear shape (B) were unaffected by loss of *Col3a1* expression at all timepoints. Myofibroblast activation, indicated by αSMA immunoreactivity, was also unaffected by *Col3a1* status (C, representative images shown in D). Macrophage density, measured as density of CD68+ cells, increased with injury but was not influenced by knockdown (E). Similarly, macrophage polarization at each healing timepoint, measured as the percentage of CD68+ cells that were iNOS−/CD206− (G), iNOS+/CD206− (H), iNOS−/CD206+ (I), and iNOS+/CD206+ (J), did not change according to reduction of *Col3a1* expression (representative images shown in F). However, percent CD68+ cells that were iNOS+/CD206− was increased in knockdown compared to wildtype tendons overall (H, p = 0.03). Scale bars are 25 μm, and quantitative data comes from regions of interest the size of representative insets. n ≥ 3 per timepoint per genotype per sex.

To understand impacts of COL3 loss on cell dynamics throughout healing, cellular composition of the tendon was explored. As anticipated, injury increased cell density (Fig. 3A, p < 0.0001 for main effect of timepoint) and nuclear circularity (Fig. 3B, p < 0.0001 for main effect of timepoint) in the healing tendon, but neither parameter was influenced by *Col3a1* knockdown at any timepoint. Myofibroblast activation was investigated with αSMA immunoreactivity, which was also increased with injury (p < 0.0001 for main effect of timepoint) and unaffected by knockdown at all healing timepoints (Fig. 3C-D).

Potential immunoregulatory contributions of *Col3a1* expression were explored by examining immunoreactivity for CD68, iNOS, and CD206. In both wildtype and knockdown tendons, the density of CD68+ cells increased with injury (p < 0.0001 for main effect of timepoint), reflecting increased macrophage density. In contrast to other tissues where COL3 has been shown to regulate immune cell phenotype and trafficking, there was no evidence to suggest *Col3a1* knockdown impacts CD68+ density in the healing tendon (Fig. 3E-F). Within CD68+ cells, the proportion of undifferentiated, M0 macrophages, which were iNOS−/CD206−, was not different between genotypes at any healing timepoint (Fig. 3G). Percent of CD68+ cells that were iNOS+/CD206−, indicating an M1, pro-inflammatory phenotype, was increased in knockdown tendons overall (p = 0.03 for main effect of genotype) independent of the healing response over time (Fig. 3H). Female knockdown tendons demonstrated this same overall increase in percent iNOS+/CD206− cells compared to wildtype (p = 0.03 for main effect of genotype), but male tendons did not (Supplemental Table 8). While this suggests COL3-dependent regulation of M1 populations, COL3 does not appear to regulate M2 populations during healing as the percentage of macrophages with M2, anti-inflammatory phenotype, indicated by iNOS/CD206+ immunolocalization, was unaffected by loss of *Col3a1* expression (Fig. 3I). Interestingly, both knockdown and wildtype tendons had large percentages of iNOS+/CD206+ cells throughout healing (Fig. 3J). Qualitatively, overall macrophage polarization profile was similar across healing timepoints. The general paucity of changes in cellular composition at each healing timepoint with *Col3a1* knockdown was consistent between female and male samples (Supplemental Table 8).

**Fig. 4.**
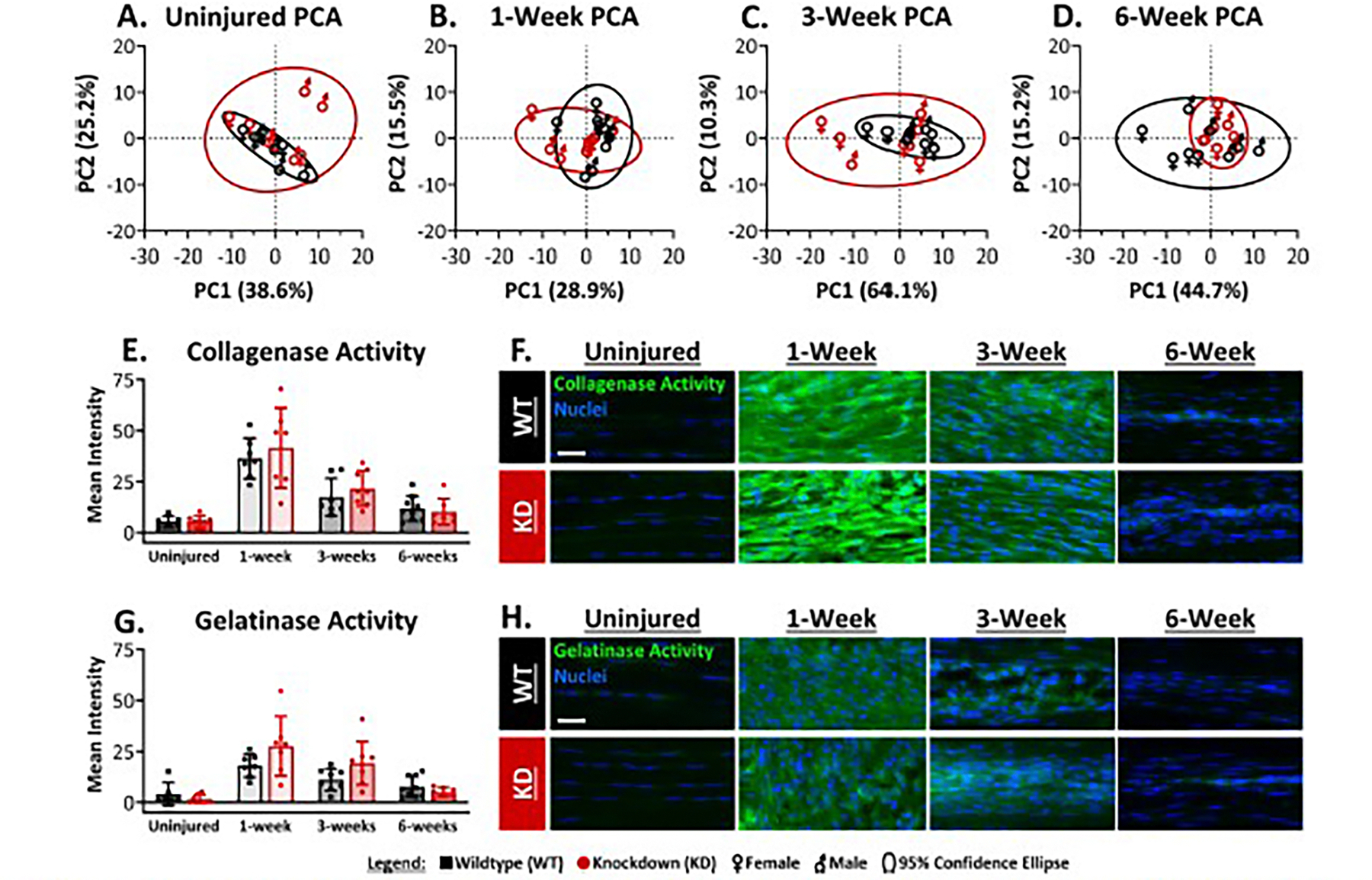
Gene expression signatures and matrix enzyme activity were unaffected by *Col3a1* knockdown. Principal component analysis (PCA) of whole tendon gene expression did not show separation between knockdown and wildtype tendons at the uninjured (A), 1-week (B), 3-week (C), or 6-week (D) timepoints, evidenced by substantial overlap of 95% confidence ellipses. Additionally, female and male tendons did not show genotype-based separation with PCA at any timepoint (A-D). Collagenase (E, representative images shown in F) and gelatinase (G, representative images shown in H) activity increased with injury but was unaffected by genotype. Scale bars are 25 μm, and quantitative data comes from regions of interest the size of representative images. n ≥ 4 per timepoint per genotype per sex for gene expression and n ≥ 3 per timepoint per genotype per sex for in situ zymography.

In the absence of notable, widespread COL3-dependent changes in myofibroblast activation and macrophage phenotype during healing, whole tendon gene expression was analyzed to capture potential knockdown-related changes in the tendon transcriptome. Whole tendon gene expression signatures were not different between knockdown and wildtype tendons at uninjured baseline or any healing timepoint. Principal component analysis (PCA) of gene expression did not demonstrate separation by genotype with ample overlap of 95% confidence ellipses for all timepoints (Fig. 4A-D). Additionally, PCA did not indicate sex-based differences in gene expression signatures between knockdown and wildtype tendons. A small proportion of genes was differentially expressed between knockdown and wildtype groups with mixed-sex analysis. Expression was decreased in knockdown compared to wildtype tendons in aggregate, but not at any specific timepoint, for six genes: *Adamts15, Eln, Mmp9, Pecam1, Tgfb3*, and *Wwtr1* (encodes TAZ, Supplemental Table 9). In uninjured tendons, *Col1a1* expression was increased in the knockdown group (Supplemental Table 9). Though no genes were differentially expressed at 1-week post-injury, expression of *Adamts2, Axin2, Dcn, Fmod, Plod2, Runx2*, and *Yap1* was decreased in knockdown compared to wildtype tendons at 3-weeks post-injury (Supplemental Table 9). At 6 weeks, knockdown tendons had increased expression of *Ltbp4* and *Yap1* (Supplemental Table 9). Still, considered collectively, there were a relatively small number of differentially expressed genes and changes in relative expression compared to wildtype for these genes were small, revealing a modest influence of *Col3a1* status on the transcriptome of the healing tendon according to multiplexed qPCR.

Given the decreased expression of *Adamts2, Adamts15*, and *Mmp9*, matrix enzyme activity was assessed with in situ zymography. Both knockdown and wildtype tendons had increased collagenase (p < 0.0001 for main effect of timepoint) and gelatinase (p < 0.0001 for main effect of timepoint) activity after injury. However, neither collagenase nor gelatinase activity were impacted by reduced *Col3a1* expression at any healing timepoint (Fig. 4E-H), regardless of sex (Supplemental Table 8). In summation, cell-focused assessment did not demonstrate significant contributions of *Col3a1* expression to cell dynamics during tendon healing.

### Reduced Col3a1 expression during tendon healing had nuanced effects on tendon mechanics

**Fig. 5.**
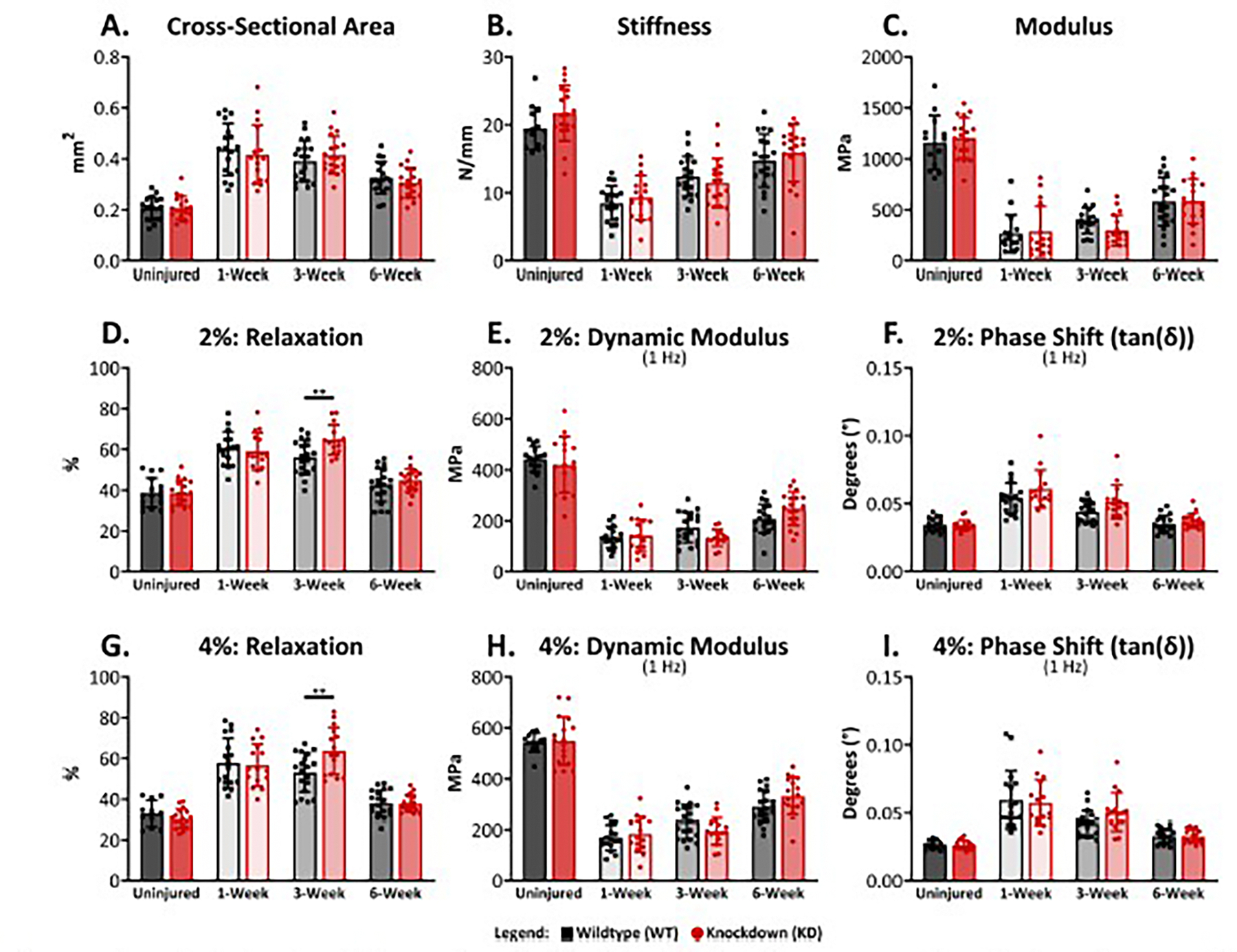
Tendon mechanics throughout healing were impacted minimally by *Col3a1* knockdown. Cross-sectional area of healing tendons was not different between genotypes (A). While both stiffness (B) and modulus (C) were affected by injury, neither demonstrated a genotype-specific difference at any healing timepoint. At 2% strain, percent relaxation (D) was increased in knockdown tendons at 3-weeks. Dynamic modulus (E) at 2% strain was unaffected by genotype at each healing timepoint. In aggregate, phase shift for the 1 Hz frequency at 2% strain was increased in *Col3a1* knockdown tendons (p < 0.01), though this difference was not noted at any healing timepoint (F). At 4% strain, percent relaxation (G) was again increased in knockdown tendons at 3-weeks. Dynamic modulus (H) and phase shift (I) were not different between genotypes at any healing timepoint at 4% strain for the 1 Hz frequency. Representative dynamic data is shown for the physiologically-relevant 1 Hz frequency; trends were generally similar across frequencies. * p < 0.05, ** p < 0.01, *** p < 0.001, and **** p < 0.0001 for multiple comparison test at each timepoint. n ≥ 8 per timepoint per genotype per sex.

To assess consequences of *Col3a1* knockdown on tissue function, tendon mechanics were assessed with elastic and dynamic viscoelastic approaches. At the tissue scale, tendon cross-sectional area increased with injury (p < 0.0001 for main effect of timepoint), but this increase was not impacted by *Col3a1* knockdown at any healing timepoint, indicating comparable scar formation in knockdown and wildtype tendons (Fig. 5A). Similarly, elastic mechanics were not changed with *Col3a1* reduction. Both tendon stiffness (p < 0.0001 for main effect of timepoint) and modulus (p < 0.0001 for main effect of timepoint) were decreased after injury. However, these injury-related changes were not influenced by loss of *Col3a1* expression at any timepoint (Fig. 5B-C), a pattern that was consistent across sexes (Supplemental Table 10).

Viscoelasticity was affected by injury and healing with slight influence from *Col3a1* knockdown. At 2% strain, knockdown tendons had increased relaxation at 3-weeks post-injury (Fig. 5D). Dynamic modulus was unaffected by knockdown (Fig. 5E) whereas phase shift was increased in knockdown tendons independent of timepoint for all frequencies at 2% strain (0.1 Hz: p = 0.04, 1 Hz: p < 0.01, 5 Hz: p < 0.01, and 10 Hz: p = 0.02 for main effect of genotype; Fig. 5F; Supplemental Table 11). This difference in phase shift was notable in male tendons where phase shift was increased in knockdown compared to wildtype tendons independent of healing timepoint for the 0.1 and 1 Hz frequencies (0.1 Hz: p = 0.03 and 1 Hz: p < 0.01 for main effect of genotype) and at 1-week post-injury for the 5 and 10 Hz frequencies (Supplemental Table 11). At 4% strain, knockdown tendons again had increased relaxation at 3-weeks post-injury (Fig. 5G). When analyzed by sex, female tendons demonstrated increased relaxation at 4% strain at 3-weeks post-injury, but male tendons did not (Supplemental Table 12). Similar to 2% strain, dynamic modulus at 4% strain was unaffected by knockdown at all frequencies and timepoints regardless of sex (Fig. 5H, Supplemental Table 12). Phase shift at 4% strain was increased in knockdown tendons collectively, independent of healing timepoint, at 10 Hz for combined sexes (p = 0.05 for main effect of genotype) and at 5 and 10 Hz for males (5 Hz: p = 0.04 and 10 Hz: p = 0.05 for main effect of genotype, Supplemental Table 12). Overall, these findings suggest modest strain-, frequency-, and sex-dependent influences of *Col3a1* expression on viscoelastic tendon mechanics, which often manifest regardless of injury status.

### Reduced Col3a1 expression during tendon healing did not influence dynamic collagen fiber alignment

**Fig. 6.**
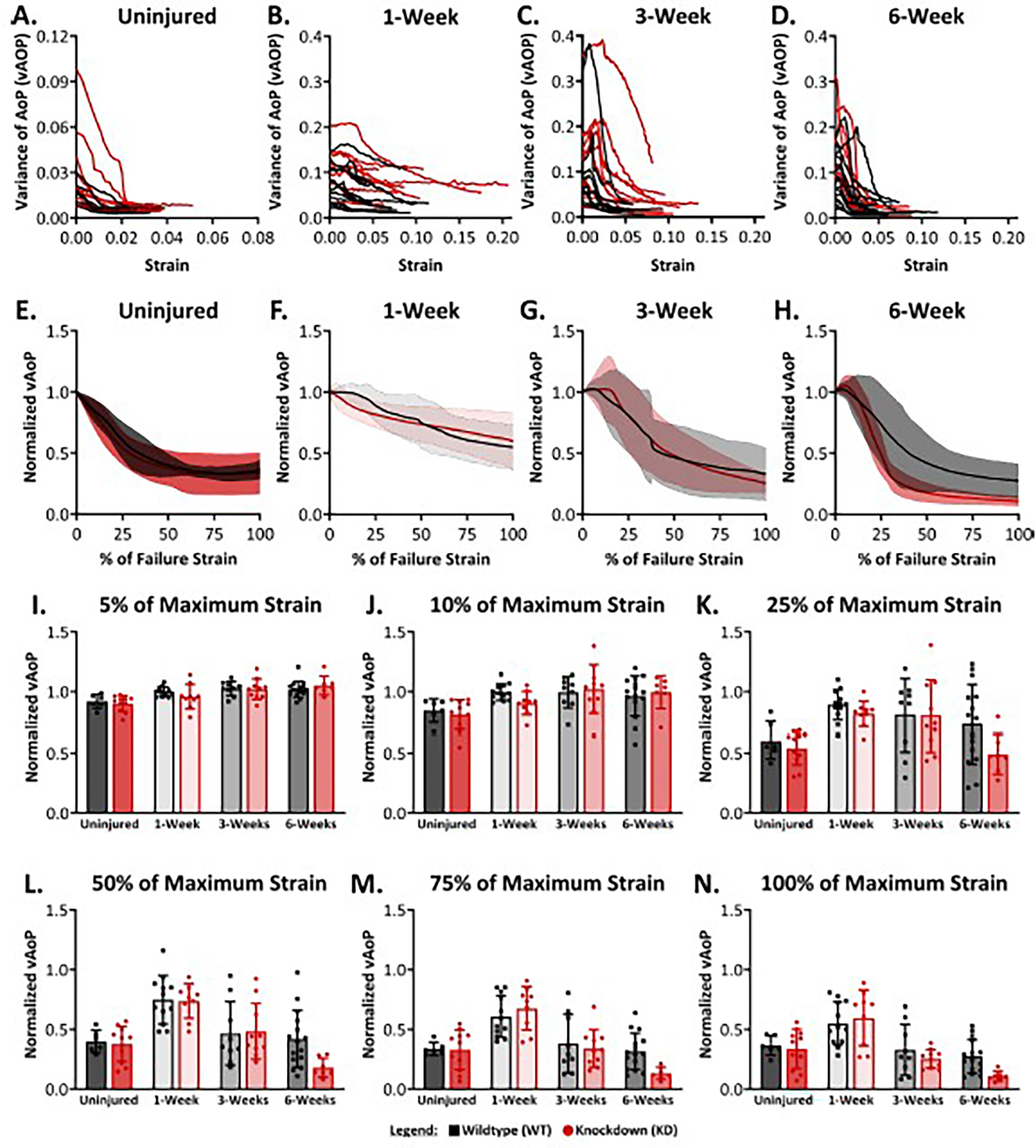
Dynamic collagen fiber alignment in healing tendons was not influenced by *Col3a1* knockdown. At each healing timepoint, variance of the angle of polarization (vAoP) values were decreased at maximum strain compared to initial values, reflecting increased alignment of the collagen fibers (A-D). Considered as a function of percent of maximum strain, average normalized vAoP was similar in knockdown and wildtype tendons across timepoints (E-H, curve represents mean and shading represents ± standard deviation). Normalized vAoP was influenced by healing timepoint but not genotype at 5 (I, p < 0.0001), 10 (J, p < 0.01), 25 (K, p < 0.001), 50 (L, p < 0.0001), 75 (M, p < 0.0001), and 100% (N, p < 0.0001) of maximum strain. n ≥ 7 per timepoint per genotype.

Effects of *Col3a1* expression on dynamic collagen fiber alignment, evaluated as changes in the variance of the angle of polarization (vAoP) of circularly polarized light reflected off the tendon midsubstance during the ramp to failure, were explored. Initial vAoP values at 0% strain varied across samples without a genotype-specific effect (Fig. 6A-D). While some samples demonstrated an initial increase in vAoP with increasing strain, all samples demonstrated an overall decrease in vAoP as maximum strain was approached, reflecting an increase in collagen fiber alignment (Fig. 6A-D). To compare changes in fiber alignment across samples, vAoP values throughout the ramp to failure were normalized to initial values at 0% strain, and strain values were considered as a percentage of maximum strain. At each healing timepoint, collagen fiber alignment in knockdown tendons was similar in trajectory and magnitude to wildtype tendons (Fig. 6E-H). At 5, 10, 25, 50, 75, and 100% of maximum strain, normalized vAoP was influenced by healing timepoint but not genotype (5%: p < 0.0001, 10%: p < 0.01, 25%: p < 0.001, 50%: p < 0.0001, 75%: p < 0.0001, and 100%: p < 0.0001 for main effect of timepoint; Fig. 6I-N). Ultimately, reduced *Col3a1* expression did not affect collagen fiber alignment during elastic loading.

## Discussion

Despite the canonical association of type III collagen and poor tendon healing, we demonstrated surprisingly little consequence of reduced *Col3a1* expression on tendon healing in young adult excisional tendon injury. In contrast to our hypothesis and prevailing dogma within the field, COL3 loss did not substantially influence matrix structure, cell behavior, or mechanical integrity during the inflammatory, proliferative, or remodeling phases of healing. While we detected nuanced differences in the effect of COL3 between females and males, including differential expression of minor collagens and other genes, the proportion of M1 macrophages, and viscoelastic mechanical properties, the overall impact on functional tendon healing was minimal in both sexes. This emphasizes the importance of advancing our understanding of mechanistic drivers of poor healing outcomes after tendon injury.

In stark contrast to what is seen in other healing tissues, *Col3a1* knockdown did not affect collagenous matrix structure, assessed at the nano- and microscales, or matrix composition, indicated by gene expression of matrix molecules, in healing tendons of young adult mice. At the nanostructure level, we did not detect a substantial influence of COL3 loss on fibril diameter despite the established role for COL3 in regulating COL1 fibril diameter [14,28–32]. Fibrils of interest in healing tendon may be too immature (< 60 nm diameter) for the influence of COL3 to be notable as COL3 may be particularly important for regulating terminal fibril diameter [14,29,31]. Even in uninjured samples, the dynamic fibril population is likely limited to small diameter fibrils [33]. COL3 facilitates increased fibril packing in cartilage, vasculature, and skin [14,31], but did not influence fibril density in healing young adult mouse tendon. This may also be attributable to investigation of relatively immature, healing tendon matrix. While previous studies have shown that collagen fibril D-band periodicity is influenced by COL3 [34, 35],we did not find differences in D-period with *Col3a1* knockdown, suggesting that molecular packing within collagen fibrils remained unaltered. COL3 also regulates fibril crosslinking [36,37]. Expression of crosslinking enzymes, *Lox, Loxl1, Loxl2*, and *Plod3*, was unaffected by *Col3a1* knockdown, but *Plod2* expression was decreased in knockdown tendons at three weeks post-injury which may indicate a subtle, temporally-specific alteration in crosslinking. However, direct assessment of collagen fibril crosslinks and crosslinking enzyme activity was not investigated, and future work could investigate differential post-transcriptional regulation of crosslinking enzymes in response to COL3 loss.

COL3 loss also had no influence on collagenous matrix density and organization during tendon healing which differs from cutaneous wound healing where COL3 loss increases matrix alignment throughout healing [12]. COL3-rich matrices are inclined to form interwoven, isotropic collagen networks [38,39]. Thus, COL3-deficient matrices are characterized as anisotropic and, though these are scar-permissive in skin, may theoretically be more favorable for tendon healing. However, COL3 loss did not alter matrix organization in healing tendon. Notably, healing matrix density was very low compared to adjacent, intact tendon, even at 6-weeks post-injury. Exploration of later healing timepoints may reveal effects of COL3 loss that emerge later in the remodeling process as the matrix continues to mature. Considered collectively, the absence of COL3-related changes in matrix structure may reveal tendon-specific matrix behaviors that should be further investigated at later healing timepoints.

Gene expression further corroborated minimal matrix changes. With whole tendon gene expression analysis, loss of *Col3a1* expression increased whole tendon *Col1a1* expression in uninjured tendons but did not cause compensatory increases in expression of genes encoding other collagen types, proteoglycans, or glycoproteins during healing. Assessment of gene expression of fibrillar collagens in the healing matrix showed timepoint- and sex-specific changes but did not demonstrate a definitive compensatory pattern. Decreased *Col11a1* at 1-week post-injury and increased *Col2a1* and *Col5a1* at 3-weeks post-injury with mixed-sex analysis may reflect nuanced changes in the COL3-deficient matrix during early phases of healing. Compared to males, female mice mounted a stronger possible compensatory fibrillar collagen response with increased *Col5a1* and *Col11a1* expression during the proliferative phase at 3-weeks post-injury. Changes in whole tendon expression of genes encoding other matrix constituents and enzymes provide more insight into COL3-related matrix changes. *Adamts2* expression was decreased at 3-weeks post-injury which may reflect reduced processing of fibrillar procollagens in the COL3-deficient proliferative matrix [40]. *Adamts15* expression was decreased with *Col3a1* knockdown independent of healing timepoint, and ADAMTS15 has versicanase activity [41]. While we did not measure expression of the gene encoding the proteoglycan versican (*Vcn*), its content is correlated with COL3 content in the vocal folds [42]. Thus, future COL3-focused investigations should probe a possible association between COL3 and versican in tendon. Of investigated proteoglycans, expression of genes encoding decorin (*Dcn*) and fibromodulin (*Fmod*) was decreased in knockdown tendons at 3-weeks post-injury, further supporting a possible relationship between COL3 content and proteoglycan content. Expression of the gene encoding elastin (*Eln*) was decreased overall in knockdown tendons which may highlight a novel association of COL3 and elastin in tendon. *Mmp9* expression was also decreased in knockdown tendons regardless of healing timepoint which contrasts with data from vascular tissue where matrix metalloproteinase-9 (MMP-9) was increased with COL3 reduction [43], suggesting tendon-specific relationships between COL3 and matrix regulating enzyme expression. In addition to types IV, V, and XI collagen, MMP-9 degrades elastin. Hence, the coordinated decrease in *Eln* and *Mmp9* expression may reflect reduced turnover of elastic fibers in the COL3-deficient matrix. Interestingly, despite decreased expression of *Mmp9*, gelatinase activity was not decreased in knockdown tendons according to in situ zymography. This may be due to post-translational regulation of enzyme activity, increased expression or activity of other gelatinases, or some combination thereof. Because our analysis was limited to changes in gene expression, direct assessment of matrix composition with proteomic approaches could further clarify alterations in the COL3-deficient matrix, but possible COL3-related matrix composition changes revealed by differential gene expression were relatively minimal and most notable at 3-weeks post-injury.

Changes in cell phenotype with COL3 loss were similarly minor. COL3 loss did reduce expression of endothelial cell marker, *Pecam1*, for knockdown tendons in aggregate. As COL3 is a main structural component of the vasculature, this may reflect decreased vasculature in knockdown tendons. In contrast to in vitro and in vivo assessment of other healing and tumor microenvironments [12,25], we found no impact of COL3 loss on myofibroblast activation by *Acta2* gene expression and αSMA immunolocalization. Importantly, αSMA synthesis may have a lower baseline during cutaneous wound healing with 20–70% of fibroblasts containing αSMA microfilaments [44,45]. We showed nearly ubiquitous αSMA content in stromal cells in the healing tendon matrix. Accordingly, high physiologic αSMA content in healing tendons may mask potential increases in αSMA due to *Col3a1* knockdown. From an immunomodulatory standpoint, *Col3a1* knockdown did not alter infiltration of antigen-presenting (*Cd80*) or T-cells (*Cd3e*). Similarly, COL3 exerted no healing-specific influence on macrophage infiltration or polarization assessed with gene expression and immunolocalization of general (*Adgre1*, CD68), pro-inflammatory (*Cd86*, iNOS), and anti-inflammatory (*Cd163* and *Cd206/Mrc1*, CD206) macrophage markers, though knockdown tendons had increased pro-inflammatory macrophage content (CD68+/iNOS+ cells) in aggregate. This differs from previous investigation of effects of recombinant humanized COL3 on macrophages in vitro and in vivo which suggested COL3 promotes anti-inflammatory macrophage polarization under inflammatory conditions [22]. Moreover, macrophage findings differ from expected tendon healing patterns [21,26,27]; we did not demonstrate a notable switch from inflammatory (CD68+/iNOS+ cells) to anti-inflammatory (CD68+/CD206+ cells) macrophage phenotype during the remodeling phase of healing which may be due to the large population of macrophages with both inflammatory and anti-inflammatory markers (CD68+/iNOS+/CD206+ cells). Future investigation of macrophage phenotype in this healing model should employ more comprehensive, single cell approaches to macrophage assessment with more phenotype markers to explicate this finding. Nevertheless, COL3 loss had little influence on cell phenotype in the healing tendon.

Changes in cell activity were also sparse. *Col3a1* knockdown tendons did have altered expression of select genes related to cell signaling. *Tgfb3* expression was decreased in knockdown tendons independent of healing timepoint. Increased *Tgfb3*, which encodes transforming beta growth factor beta 3 (TGF-β3), is associated with improved tendon healing [46] and may increase production of COL1 and COL3 [47,48]. Hence, the observed decreased expression of *Tgfb3* may reflect altered regulation of relative COL1 and COL3 production. Further, increased expression of the gene encoding latent transforming growth factor beta binding protein 4 (*Ltbp4*) at 6-weeks post-injury may reflect altered TGF-β sequestration and signaling. Still, these changes in TGF-β*-*related gene expression were not structurally or mechanically consequential. Decreased expression of *Yap1*, the gene that encodes Yes-associated protein 1 (YAP), at 3- and 6-weeks post-injury and *Wwtr1*, the gene that encodes WW domain containing transcription regulator 1 (TAZ), regardless of healing timepoint may underscore altered mechanosensation with COL3 loss. Since COL3 deficient fibroblasts have altered mechanosensation through YAP and TAZ nuclear translocation [12], decreased *Yap1* and *Wwtr1* expression may be a consequence of altered signaling through the mechanoregulatory Hippo signaling pathway. While *Col3a1* knockdown in healing tendons of young adult mice did not change expression of investigated integrin-related genes, data from skin suggests COL3 loss increases integrin α11 expression [12]. Since these changes in cell signaling were only investigated through gene expression, further investigation into changes in tendon cell mechanobiology with *Col3a1* knockdown should directly assess integrin content and behavior as well as YAP and TAZ nuclear translocation. Additionally, unbiased approaches to investigating gene expression changes may highlight other COL3-related changes in cell activity.

The minimal influence of *Col3a1* knockdown on tissue mechanics is consistent with nominal matrix and cell changes. Knockdown tendons had increased viscosity, indicated by increased relaxation and phase shift. At 3-weeks post-injury, relaxation was increased in knockdown samples at both 2 and 4% strain. This may reveal a role for COL3 in regulating tissue viscoelasticity during the proliferative phase of healing. Phase shift was increased at 2% (0.1, 1, 5, and 10 Hz) and 4% (10 Hz) strain, independent of healing timepoint, which may indicate nuanced, injury-independent viscoelastic mechanical compromise due to COL3 loss. This compromise was also notable in male mice during early healing at 1-week post-injury (2% strain, 5 and 10 Hz) which is consistent with the importance of COL3 in establishing provisional matrix mechanical integrity during early healing. More significant effects of *Col3a1* knockdown in injured young adult male tendons are consistent with effects of COL3 reduction being more significant in aged male mice [49]. However, in general, *Col3a1* knockdown did not affect female and male tendons differently, and knockdown tendons were otherwise not mechanically different from wildtype tendons. COL3 loss was not associated with changes in elastic mechanical behavior, and collagen fiber alignment throughout loading was not different between knockdown and wildtype tendons, corroborating the paucity of collagen fiber and elastic mechanical differences. These findings are surprising given the well-established relationship between COL3 content and tissue stiffness [15,50–56]. While injured tendons were stamped with a biopsy punch to remove much of the uninjured tissue, portions of the uninjured tendon adjacent to the injury may remain which could influence tissue-scale mechanical properties. Still, similar studies investigating knockdown of other matrix constituents, including fibrillar collagens, demonstrated detectable mechanical changes with this injury model [57,58], emphasizing the intriguing nature of the minimal impact of *Col3a1* knockdown on mechanical properties. Targeted mechanical assessment of isolated injury matrix could provide additional insight into effects of COL3 loss on recovery of mechanical properties in the healing tendon matrix.

Ultimately, these findings challenge the notion that COL3 deposition during the early stages of healing drives poor outcomes after tendon injury, although it remains possible that COL3 could impact maturation of the healing matrix at later stages of healing beyond 6 weeks. In skin, COL3 deficiency substantially altered matrix and cell behavior during healing [12,25]. Differential healing responses to COL3 loss in skin and tendon have many possible causes. Skin has much higher COL3 content and a basketweave-structured matrix at baseline which may warrant a stronger COL3 healing response. Additionally, contractile matrix and cell behavior is fundamental to cutaneous skin healing after excisional injury. In mouse patellar tendon, contractile behavior may be less important for healing excisional injuries. Exploration of other injury models and later healing timepoints will provide additional insights into COL3-dependent healing mechanisms. Possible compensatory production of other matrix proteins may mitigate COL3 loss; unbiased transcriptomic and proteomic approaches may reveal compensation mechanisms not detected with our approach. Furthermore, because *Col3a1* expression [59] and compensatory mechanisms may be altered with age, effects of *Col3a1* knockdown in developing and aged tendons should also be explored. Defining changes in the COL3-deficient healing matrix throughout the lifespan will inform future studies that seek to understand contributions of COL3 to age-specific tendon healing. This will eventually progress treatment of tendon injuries in the growing population of patients with reduced COL3 associated with advanced age [60], menopause [61], tobacco use [62], and vascular Ehlers Danlos syndrome.

In conclusion, our study demonstrates a lack of mechanistic impact of COL3 loss on tendon healing in young adult mice that is consistent across sexes. This challenges the historical association of COL3 with poor healing outcomes and underscores the importance of defining the composition of the healing tendon matrix to discern mechanisms of poor tendon healing.

### Experimental procedures

#### Animal use & study design

Animal use was approved by the University of Pennsylvania Institutional Animal Care and Use Committees (protocol number 803267) and was consistent with the recommendations in the Guide for the Care and Use of Laboratory Animals of the National Institutes of Health. We leveraged a C57BL/6 mouse model of global, tamoxifen-inducible (Rosa26-CreERT2) *Col3a1* knockdown [12], using mixed-sex mice with *Col3a1* wildtype (WT, *Col3a1*^WT/WT-ERT2/ERT2^) and *Col3a1* knockdown (KD, *Col3a1*^Flox/Flox-ERT2/ERT2^; loxP sites flanking Exon 2) genotypes. Because of sex-specific differences in tendon healing [63], collagen synthesis [64], and COL3 levels [65], both female and male mice were included with sufficient power to examine sex-specific effects of COL3 loss. Mice were housed in a controlled facility with a 12-hour light/dark cycle, chow and water ad libitum, and regular pathogen surveillance. All mice received a single tamoxifen injection (200 mg/kg; T5648, Sigma-Aldrich, St. Louis, MO) at postnatal day 89 (p89). Then, at p90, mice were randomly assigned to injury groups and received bilateral patellar tendon biopsy punch (0.75 mm diameter) surgery to create excisional wounds [66]. Mice were randomly assigned to sacrifice at 1-, 3-, or 6-weeks post-injury to examine the inflammatory (early), proliferative (middle), and remodeling (late) healing phases, respectively [67,68]. Mice randomly assigned to uninjured groups also received tamoxifen injection at p89 and were collected at the 3-week post-injury timepoint to maximize comparability to all healing timepoints. After humane euthanasia, performed according to guidelines set forth by the American Veterinary Medical Association, patellar tendons were collected bilaterally by investigators blinded to mouse genotype for assessment of matrix, cell, and mechanics outcomes (Supplemental Figure 1A-G).

#### Tendon matrix assessment

##### Tendon matrix COL3 content (n ≥ 3 per timepoint per genotype per sex, supplemental figure 1A)

After harvest, mouse knees were fixed in Prefer (Anatech, Battle Creek, MI) for 24 h, then optically cleared in 30% sucrose (Sigma-Aldrich, St. Louis, MO) in 1X phosphate buffered saline (weight by volume) for 24 h. Patella-patellar tendon-tibial insertion complexes were dissected, cryoembedded in Tissue-Tek^®^ O.C.T. Compound (Sakura, Torrance, CA), and sectioned at 8 μm in the anterior-posterior plane with cryofilm (Cat Cryofilm type 2C (10), Section-Lab, Yokohama, Japan) [69,70]. Four cryofilm sections from each sample were adhered to glass slides with 1% chitosan in acetic acid and dried [69,70] for immunostaining (2 sections) and zymography (2 sections). One section per sample was post-fixed with 4% paraformaldehyde, incubated with pepsin (0.5 mg/mL in 0.01 N HCl; Worthington, Lakewood, NJ) for antigen retrieval, and immunolocalized with fluorescent substrates for COL3 (goat anti-mouse type III collagen and donkey anti-goat Alexa Fluor^™^ Plus 647, Supplemental Table 1) before imaging with a Leica SP-8 confocal microscope (20X, water immersion objective; Leica Microsystems, Wetzlar, Germany). Quantification of fluorescent intensity was performed for a 50 μm × 100 μm region at the center of the wounded area, or field of view for uninjured samples, in Fiji (v2.16.0) [71].

##### Collagenous tendon matrix production (n = 5 per healing timepoint per genotype per sex, supplemental figure 1B)

To assess potential compensatory changes in matrix production with *Col3a1* knockdown, expression of other fibrillar collagens in the healing matrix was examined for 1-, 3-, and 6-week post-injury samples. Knees were fixed in RNase-free 4% paraformaldehyde (Electron Microscopy Sciences, Hatfield, PA) for 5 h, cryoembedded in Tissue-Tek^®^ O.C.T. Compound, and sectioned at 40 μm in the transverse plane. Healing tissue was microdissected under 10X magnification with a 25 G needle. Tissue was digested and de-crosslinked (Proteinase K, Zymo, Irvine, CA), and RNA was isolated (*Quick*-RNA Microprep Kit, Zymo, Irvine, CA). Quality of isolated RNA was assessed for 8 randomly selected samples with Agilent BioAnalyzer (Agilent, Santa Clara, CA); RNA integrity numbers (RIN) indicated acceptable RNA quality (7.55 ± 0.76 (mean ± standard deviation)) [72]. RNA was converted to cDNA (High-Capacity cDNA Reverse Transcription Kit, Thermo, Waltham, MA), preamplified for selected probes (TaqMan, Thermo, Waltham, MA), and assessed with qPCR (QuantStudio^™^ 6, Thermo, MA) for housekeeper (*Abl1*) and fibrillar collagen genes (*Col1a1, Col1a2, Col2a1, Col3a1, Col5a1*, and *Col11a1*; Supplemental Table 2). ΔCt values were calculated as the difference of Ct values for the housekeeper (*Abl1*) and Ct values for collagens of interest (ΔCt = Ct_*Abl1*_ – Ct_*Fibrillar Collagen*_).

##### Tendon matrix collagen fibril structure (n = 4 per timepoint per genotype per sex, supplemental figure 1C)

Dissected uninjured as well as 3-week and 6-week post-injury patellar tendons were fixed in Karnovsky’s fixative, post-fixed in 1% osmium tetroxide, dehydrated in increasing concentrations of ethanol, and embedded in Epon (Embed 812, Electron Microscopy Services, Hatfield, PA) [73]. Embedded tendons were sectioned at 80 to 100 nm in the transverse plane with an ultramicrotome and post-stained with UranyLess EM Stain (Electron Microscopy Services, Hatfield, PA) then 1% phosphotungstic acid. After imaging with a JEOL 1010 transmission electron microscope (60,000X; JEOL USA, Peabody, MA), custom software (MATLAB, R2024a, Mathworks, Natick, MA) assessed fibril diameter for 1,000 randomly selected collagen fibrils [74] and fibril density for ten 750 nm × 750 nm regions of interest per sample. Analyzed fibrils were in the healing region for injured samples or center of the tendon for uninjured samples. Fibril structure for 1-week samples was not assessed due to fibril immaturity in the early healing matrix.

##### Tendon matrix collagen fibril D-period (n = 2 per timepoint per genotype per sex, supplemental figure 1D)

Dissected patellar tendons from uninjured, 3-week, and 6-week groups were fixed overnight in 10% neutral buffered formalin (Astral Diagnostics, Logan Township, NJ), cryoembedded in Tissue-Tek^®^ O.C.T. Compound, and sectioned at 20 μm in the anterior-posterior plane. Collagen fibrils were imaged using a Bioscope Catalyst atomic force microscope (Bruker, Billerica, MA) operated in tapping mode in air with a VTESPA 300 cantilever tip (Bruker, Billerica, MA). For injured samples, images were captured near the injury site. For each sample, three 2 × 2 μm images were captured with a scan speed of 1 Hz at 512 × 512 resolution. Fibril D-period was measured from manually selected fibrils in each image using a custom MATLAB script [75] and averaged across each sample.

##### Tendon matrix collagen fiber structure (n ≥ 3 per timepoint per genotype per sex, supplemental figure 1E)

Harvested knees were fixed in 4% paraformaldehyde for 6 h, then patella-patellar tendon-tibial insertion complexes were dissected and fixation was continued overnight. After rinsing, nuclei were stained with SYTOX^™^ Green (1:1,000; Thermo, Waltham, MA) for 24 h. Then, whole tendon complexes were optically cleared with a modified version of SeeDB (Supplemental Table 3) [76,77] and coverslipped in 115% fructose (weight by volume; Sigma-Aldrich, St. Louis, MO) with 0.2% α-thioglycerol (volume by volume; Sigma-Aldrich, St. Louis, MO) in deionized water. Tendons were imaged with a Leica SP8 confocal/multiphoton microscope (20X, water immersion objective) with the Coherent Chameleon Ultra II Ti:Sapphire laser (Coherent, Saxonburg, PA) tuned to 910 nm. Forward second harmonic generation (fSHG) signals were collected on a nondescanned detector configured to capture 455 nm wavelengths. Scan speed was set at 400 Hz, and line average was set to 3. Quantification of matrix disorganization, as the circular standard deviation of fiber alignment according to OrientationJ (v2.0.6) [78], and density, as the mean intensity of the fSHG signal, was performed for a 50 μm × 100 μm region at the center of the wounded area, or field of view for uninjured samples, in Fiji [71]. Due to substantial differences in matrix density after wounding, laser power was set at 12% for uninjured and 15% for injured samples; thus, comparisons of matrix density were performed for injured samples exclusively.

#### Tendon cell assessment

##### Tendon cell phenotype (n ≥ 3 per timepoint per genotype per sex, supplemental figure 1A)

Tissue sections stained for COL3 (See Methods: Tendon Matrix COL3 Content) were also immunolocalized with fluorescent substrates, after antigen retrieval with pepsin (0.5 mg/mL in 0.01 N HCl), for conjugated anti-mouse α-smooth muscle actin-Cy3 (Supplemental Table 1) and counterstained with SYTOX^™^ Green for nuclei (1:100,000). Slides were imaged with a Leica SP-8 confocal microscope (20X, water immersion objective). Using Fiji [71], cell density, nuclear circularity (0 indicates line, 1 indicates perfect circle), and mean intensity of the αSMA signal were quantified for the same 50 μm × 100 μm region at the center of the wounded area, or field of view for uninjured samples, used for COL3 content quantification.

To explore macrophage phenotype, tissue sections were post-fixed with 4% paraformaldehyde; incubated with Proteinase K (diluted 1:1,000 in TE Buffer; Thermo, Waltham, MA) for antigen retrieval; immunolocalized with fluorescent substrates for CD68 (rat anti-mouse CD68 and donkey anti-rat Alexa Fluor^™^ 488), inducible nitric oxide synthase (iNOS, rabbit anti-mouse iNOS and donkey anti-rabbit Alexa Fluor^™^ 555), and CD206 (goat anti-mouse CD206 and donkey anti-goat Alexa Fluor^™^ 647, Supplemental Table 1); and counterstained with Hoechst 33342 (1:1,000; Thermo, Waltham, MA). Macrophage-stained slides were imaged with a Zeiss Axioscan (20X; Zeiss, Oberkochen, Germany). Using a custom software (MATLAB), CD68+ cell density was quantified and averaged for two 100 μm × 200 μm regions at the center of each wounded area, or field of view for uninjured samples. For injured samples, the population of CD68+ cells was further assessed for percentage of iNOS+ and CD206+ cells.

##### Tendon cell gene expression (n ≥ 4 per timepoint per genotype per sex, supplemental figure 1F)

Immediately after sacrifice, whole tendons were dissected, incubated in RNA*later*^™^ (Thermo, Waltham, MA) at 4°C overnight, and then stored at −80°C. For RNA isolation, samples were thawed, homogenized in QIAzol^™^ Lysis Reagent (QIAGEN, Venlo, Netherlands), and processed according to manufacturer’s protocols (Direct-zol RNA Microprep, Zymo, Irvine, CA). RNA quality for 8 randomly selected samples was acceptable (RIN 7.67 ± 0.53 (mean ± standard deviation), Agilent BioAnalyzer) [72]. RNA was converted to cDNA (High-Capacity cDNA Reverse Transcription Kit) and pre-amplified for selected genes (TaqMan probes, Supplemental Table 4). Pre-amplified cDNA was assessed with qPCR for 92 genes (96.96 Dynamic Array IFC, BMK-M-96.96, Standard BioTools, San Francisco, CA), including genes encoding matrix composition, matrix enzyme, mechanobiology, cell phenotype, and cell signaling proteins (Supplemental Table 4). ΔCt values were calculated as the difference of average Ct values for housekeeping genes and Ct values for genes of interest (*18S, Abl1*, and *Rps17*; ΔCt = (Ct_*18S*_ + Ct_*Abl1*_ + Ct_*Rps17*_)/3 – Ct_*Gene of Interest*_).

##### Tendon cell matrix metalloproteinase activity (n ≥ 3 per timepoint per genotype per sex, supplemental figure 1A)

Prefer-fixed cryosections from uninjured as well as 1-, 3-, and 6-week post-injury samples (see Methods: Tendon Matrix COL3 Content) were rinsed twice with filtered 1X phosphate buffered saline to remove Tissue-Tek^®^ O.C.T. Compound for in situ zymography. Fluorescein-conjugated DQ^™^ collagen type I (D12060, Thermo, Waltham, MA) or DQ^™^ gelatin (D12054, Thermo, Waltham, MA) was diluted (0.1 mg/mL) in a buffer consisting of 50 mM Tris–HCl, 150 mM NaCl, 5 mM CaCl_2_, and 0.2 mM NaN_3_ (pH 7.6). Tissue sections were incubated with diluted collagen or gelatin solutions in a light-blocking, humidified chamber for 1 h at room temperature, rinsed, fixed with 4% paraformaldehyde, and mounted with Fluoromount-G (Thermo, Waltham, MA) with DAPI (Thermo, Waltham, MA). Fluorescence was detected with a Zeiss Axioscan (20X). Mean fluorescent intensity, as an indicator of enzyme activity, was quantified in Fiji [71] for a 100 μm × 200 μm region at the center of each wounded area, or field of view for uninjured samples.

#### Tendon mechanics assessment

##### Tendon viscoelastic and elastic mechanics (n ≥ 8 per timepoint per genotype per sex, supplemental figure 1G)

Patella-patellar tendon-tibia complexes were dissected. Tendons were stamped with a biopsy punch (2 mm diameter) to create a dog bone shape with 0.75 mm width centered on the wound, or midline for uninjured samples [57,66]. The tendon midsubstance cross-sectional area was measured with a custom laser-based system [79] and stained with Verhoeff’s stain at 0.5 mm proximal and distal to the center of the wound, or longitudinal center of the tendon for uninjured samples. After potting the tibia in polymethyl methacrylate, the complex was mounted in a materials testing machine (5848, Instron, Norwood, MA) and tested with a protocol including preconditioning (10 sinusoidal cycles with 0.25% strain amplitude centered at 1% strain), 5 min of unloaded rest (0% strain), viscoelastic assessment at 2% and 4% strain (ramp to strain; hold for 10 min; 10 sinusoidal cycles with 0.625% strain amplitude at 0.1, 1, 5, and 10 Hz), 5 min of unloaded rest (0% strain), and elastic assessment with a quasi-static ramp to failure (0.1% strain per second with image capture at 2 Hz; BFS-U3–51S5P-C, FLIR Integrated Imaging Solutions, Richmond, BC, Canada). Testing was performed in a 1X phosphate buffered saline bath at 37°C. Mechanical properties were calculated using load, displacement, and time data, and elastic modulus was determined with a custom optical strain tracking software (MATLAB).

##### Tendon dynamic collagen fiber alignment (n ≥ 7 per timepoint per genotype, supplemental figure 1G)

Circularly polarized light reflected off the tendon midsubstance was captured with reflectance-mode quantitative polarized light imaging. The variance of the angle of polarization (vAoP) indicated collagen fiber alignment, where a lower variance indicated higher fiber alignment across the tissue [80]. Using a custom software, vAoP for the central third of the tendon between the Verhoeff’s stain lines was assessed during the ramp to failure for samples with adequate light reflection. Variance of the angle of polarization was normalized to the value at 0% strain to capture changes with loading and statistically compared at 5, 10, 25, 50, 75, and 100% of maximum strain.

### Statistics

Data were evaluated for normality (Shapiro-Wilks normality tests), and samples were excluded from analysis when consistent statistical outliers were identified (≥ |2.2*interquartile range| established a priori). Most quantitative data were compared with two-way ANOVAs (healing timepoint, genotype) with Šídák’s multiple comparison tests to discern genotype-based differences at each healing timepoint (GraphPad Prism, v10, Boston, MA). When pertinent, p values for main effects of healing or timepoint are included in the text. Data for fibrillar collagen expression in the healing matrix were assessed with Student’s two-tailed *t*-tests comparing genotypes at each timepoint to ensure valid comparisons within qPCR plates. Multiplexed qPCR gene expression profiles were compared with principal component analysis (MATLAB). Data were assessed with female and male data combined (mixed-sex, presented in figures) as well as female and male data separately (presented in supplemental tables). Significance was set at p ≤ 0.05, and data are presented as mean ± standard deviation.

## Supplementary Material

Suppl Figure 1

Suppl Figure 2

Suppl Figure 3

Supp Table 2

Supp Table 3

Supp Table 1

Supp Table 4

Supp Table 6

Supp Table 7

Supp Table 8

Supp Table 9

Supp Table 10

Supp Table 11

Supp Table 12

Supp Table 5

Supplementary material associated with this article can be found, in the online version, at doi:10.1016/j.matbio.2026.102012.

## Data Availability

Data will be made available on request.
