# Supplementary material for: Loss of type III collagen has limited effect on tendon healing in young adult mice
Source: Matrix Biol. Author manuscript; Available in PMC 2026 Aug 8. (PMC13451987; doi:10.1016/j.matbio.2026.102012)
Supplement: Supplementary file 1 [file NIHMS2197763-supplement-Suppl_Figure_1.docx]

**Supplemental Figure 1. Patellar tendons were collected bilaterally for matrix, cellular, and mechanical assessment.** Immunolocalization of COL3, αSMA, and macrophage markers as well as assessment of matrix enzyme activity was performed on anterior-posterior plane cryosections of patella-patellar tendon-tibia complexes (A, n ≥ 3 per timepoint per genotype per sex). For fibrillar collagen gene expression, patella-patellar tendon-tibia complexes were fixed under RNAase-free conditions and cryoembedded; samples were sectioned in the transverse plane and healing tissue was microdissected and processed for qPCR (B, n = 5 per timepoint per genotype per sex for injured timepoints). Collagen fibril parameters were assessed with transmission electron microscopy of samples embedded in Epon and sectioned in the transverse plane (C, n = 4 per timepoint per genotype per sex, 1-week timepoint not assessed) and with atomic force microscopy of anterior-posterior plane cryosections (D, n = 2 per timepoint per genotype per sex, 1-week timepoint not assessed). Collagen fiber parameters were assessed with second harmonic generation imaging of optically cleared samples in the anterior-posterior plane (E, n ≥ 3 per timepoint per genotype per sex). For whole tendon gene expression, samples were preserved in RNA*later*TM until RNA isolation, cDNA conversion, and pre-amplification for assessment of gene expression with multiplexed qPCR (F, n ≥ 4 per timepoint per genotype per sex). Dynamic viscoelastic and elastic mechanical assessment was performed on samples dissected and prepared after one freeze thaw cycle (G, n ≥ 8 per timepoint per genotype per sex). Reflectance-mode quantitative polarized light image capture allowed for measurement of the variance of the angle of polarization during elastic mechanical assessment to quantify dynamic collagen fiber alignment (G, n ≥ 7 per timepoint per genotype).
